# Acute Abdomen in Pregnancy due to Idiopathic Chylous Ascites

**DOI:** 10.1155/2024/8898451

**Published:** 2024-02-29

**Authors:** Claudia Epelde, Fátima Saravia, Mónica Aguinaga, Ane Toledo, Arantza Lekuona, Mikel Gorostidi

**Affiliations:** Department of Obstetrics and Gynecology, Donostia University Hospital, San Sebastián, Basque Country, Spain

## Abstract

Chylous ascites results from the leakage of lymph rich in lipids into the peritoneal cavity and represents an exceedingly rare event in the course of pregnancy. While there are numerous documented instances of this pathology manifesting with hypogastric or diffuse abdominal pain, our report highlights a unique presentation involving a 35-week pregnant woman experiencing severe epigastric pain unrelated to pregnancy-induced hypertension or other gastrointestinal disorders. Major acute obstetric pathologies were ruled out, and there was no evidence of fetal distress. Due to uncontrolled pain with an unidentified etiology and an unfavorable Bishop score, an urgent cesarean section was performed. A copious amount of milky fluid was observed during the surgery, subsequently confirmed as chylous in nature. Both the newborn and the mother had positive outcomes postsurgery. Although it is usually a benign condition, it is important for healthcare professionals to be aware of this entity in order to provide timely medical care and administer appropriate treatment.

## 1. Introduction

Chylous ascites (CA) is a rare form of ascites that results from the exudation of lipid-rich lymph into the peritoneal cavity. It can manifest as abdominal distension or pain or be completely asymptomatic. Underlying etiologies of CA have been classified as traumatic, congenital, infectious, neoplastic, postoperative, cirrhotic, or cardiogenic. Nearly two-thirds of all CA cases in developed countries are associated with neoplasms (solid organ malignancy, lymphoma, lymphangiomyomatosis, carcinoid tumors, and Kaposi sarcoma) or cirrhosis. Diagnosis is established based on clinical presentation and analysis of ascitic fluid [1].

Chylous ascites has rarely been associated with pregnancy. Only a few cases have been reported, and a clear etiology has been found in a small number of these. Most of the published cases have presented with hypogastric or generalized abdominal pain, fever, or hypertensive disorders, or as a finding during a cesarean performed for other reasons [2–12].

The purpose of this article is to describe a case of chylous ascites during pregnancy and to provide an update on this topic.

## 2. Case Presentation

A 35-year-old G2T1P0A0L1 woman at 35 + 4 weeks of gestation, without any known comorbidities, presented to the emergency department with sudden epigastric abdominal pain. The pain was constant with episodes of exacerbation, and there was no improvement despite oral analgesics. There were no amniorrhea, uterine contractions, metrorrhagia, or other symptoms suggestive of preeclampsia. Prior to this episode, she had had normal prenatal check-ups. She had a history of epigastralgia, which had been investigated in 2017 with normal gastroscopy and colonoscopy. There were no noteworthy family history records.

**Figure 1:**
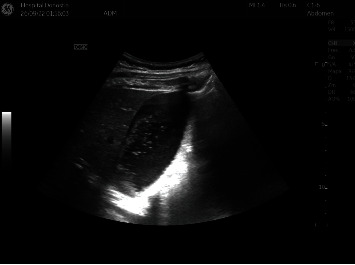
Abdominal ultrasound: distended gallbladder without clear ultrasound signs of acute cholecystitis and a minimal perihepatic fluid pocket were observed.

During her stay in the emergency department, the patient remained hemodynamically stable. She remained normotensive and normocardial. She had no fever or increased respiratory rate. The boundary sign of abdominal wall with compression was absent at admission. In the gynecological examination, no accompanying pathological findings suggestive of acute obstetric pathology that could compromise fetal well-being were observed. Cardiotocography showed no signs of fetal distress, and the mother presented very scarce irregular, ineffective contractions. Blood tests were performed, including liver and renal profiles, and were normal (glucose 103 mg/dL, hemoglobin 11.7 g/dl, leukocytes 9150/*μ*L, C reactive protein 1.1 mg/L, GPT 15 U/L, GOT 23 U/L, FA 256 mg/dL, GGT 10 U/L, lipase 17 U/L, amylase 42 U/L, total bilirubin 0.2 mg/dL, creatinine 0.52 mg/dL, urea 20 mg/dL, calcium 9 mg/dL, proteins 5.4 g/dL, Na 138 mEq/L, K 3.9 mEq/L, no analytical findings of coagulopathy). Urinalysis resulted normal. The patient required a large amount of IV analgesics for pain management (1 vial of dipyrone 2 g IV, 1 vial of acetaminophen 1gr IV, 1 vial of omeprazole 40 mg IV, 1 vial of scopolamine butyl bromide 20 mg IV, and 6 cc IV of morphine chloride). To identify the cause of the pain, an abdominal ultrasound was performed. Only a distended gallbladder without clear ultrasound signs of acute cholecystitis and a minimal perihepatic fluid pocket were observed (Figure 1). Any other infectious origin of the pain was ruled out. The general surgery department was called upon, and based on the findings from the conducted tests, they excluded the possibility of a digestive origin of the abdominal pain.

**Figure 2:**
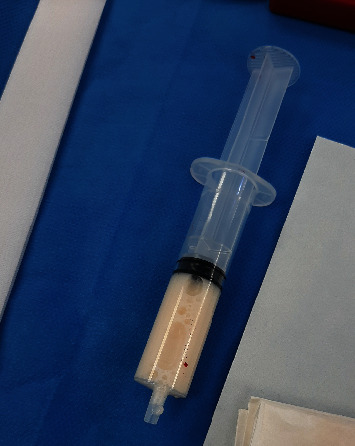
Milky fluid was evacuated from the upper abdomen.

Due to poor pain control with an unidentified etiology and an unfavorable Bishop score, an urgent cesarean section was performed. Upon entering the peritoneal cavity, approximately 500 cc of milky fluid was evacuated from the upper abdomen (Figure 2). A live newborn weighing 3140 g was born with Apgar scores of 8 and 5 at 1 and 5 minutes, respectively. The amniotic fluid was clear, and the placenta showed no signs of detachment. General surgery was consulted for intraoperative assessment. No abnormalities were observed during the intervention: the uterus and adnexa appeared normal, the loops of small intestine had good coloration with present peristalsis and moderate dilation, and the appendix had a normal appearance. The estimated blood loss during the surgery was 500 cc, and only 10 units of oxytocin IV bolus were required to facilitate placental delivery and uterine involution. The rest of the surgery proceeded without complications. After the procedure, samples were sent for microbiological and cytochemical analysis. Antibiotics were not administered postsurgery, as the milky appearance rather than purulent nature of the peritoneal fluid raised a strong suspicion of chylous ascites. Finally, the cultures turned out negative, and a triglyceride level of 1580.20 mg/dL was recorded, consistent with chylous ascites.

The postoperative course was favorable with complete resolution of the pain experienced upon admission. A high-protein, low-fat diet was initiated along with medium-chain triglyceride supplementation and a somatostatin infusion. Postcesarean analytical monitoring showed normal results, with no hepatic or renal abnormalities. The patient was discharged from the hospital on the 4th postoperative day after an ultrasound examination showed no evidence of free fluid in the peritoneal cavity. Given the satisfactory progress, it was considered to be a case of spontaneous idiopathic chylous ascites.

Following hospital discharge, the patient attended several follow-up appointments with both the gynecologist, as well as with gastroenterology department. A CT scan was performed, and the presence of H. pylori was ruled out through the breath test. The patient did not experience a similar episode and was well recovered from both a digestive and gynecological perspective. Therefore, she was discharged from outpatient follow-up 1 year after the episode.

## 3. Discussion

Chylous ascites is a rare form of ascites that results from the leakage of lipid-rich lymph into the peritoneal cavity. This case report presents an uncommon presentation of this condition during pregnancy, which is itself a very rare finding. This article is aimed at shedding light on this disease, for which there is scarce published literature.

The most common cause is malignancy, with lymphoma being the most frequently associated tumor. Other causes may be related to liver disease, abdominal trauma, congenital conditions (such as lymphatic dysplasia), inflammatory causes (vasculitis), infectious diseases (filariasis, schistosomiasis), or postoperative complications (especially following abdominal surgeries or pelvic and para-aortic lymphadenectomy) [13].

The lymphatic system transports lymph throughout the body. Lymph is rich in proteins, triglycerides, and lymphocytes, so severe leaks can lead to hypoproteinemia due to the loss of albumin, fibrinogen, and immunoglobulins. Depletion of fat reserves and fat-soluble vitamins can also be observed. From a cellular perspective, lymphocytes represent 95% of the cellular content of chyle, which can lead to lymphopenia and an increased risk of infections.

A detailed medical history and physical examination are important for diagnosing chylous ascites. The most common symptom is abdominal distention (81%), followed by pain or peritonitis in 11% of cases [1].

In pregnancy, chylous ascites is an extremely rare finding associated with nonspecific symptoms. Cases have been described that present with abdominal pain, fever [3, 4], severe hypertensive disorders of pregnancy [2, 6], and even asymptomatic patients [9] with an incidental finding during cesarean section.

A Spanish team described a case of diffuse abdominal pain associated with leukocytosis in a pregnant woman in the second trimester who, due to the clinical worsening of the patient, was suspected of having peritonitis due to perforation of hollow abdominal viscera. A diagnostic laparoscopy was performed, where the presence of chylous fluid in the abdominal cavity was observed without other pathological findings [10].

Burgos Luna et al. [6] and Apikotoa and Wijesuriya [4] describe cases of chylous ascites in pregnant women, where the main symptomatology was diffuse abdominal pain. In the first case, it was associated with hypertensive encephalopathy; in the second case, acute complicated appendicitis was suspected.

Yang et al. [8] described a case of a pregnant woman with fever and epigastric abdominal pain who, and although no analytical or ultrasound abnormalities were observed, due to the patient's poor general condition, a cesarean section was performed, and a chylous ascites was discovered. This was the first case of hypertriglyceridemia-induced acute pancreatitis with normal pancreatic enzymes.

Two cases of acute abdominal pain and distension occurring in the immediate postpartum period following a eutocic delivery have also been described, which required exploratory laparotomy revealing milky ascitic peritoneal fluid [5, 9].

We have identified only one documented case of chylous ascites during gestation secondary to an organic pathology, caused by an aggressive mesenteric fibromatosis [12] Table 1 describes the cases of chylous ascites in pregnant women reported to date.

Due to the ambiguity of associated symptoms, performing a paracentesis has been described as the gold standard technique for diagnosis, typically yielding ascitic fluid with a milky appearance and triglyceride levels > 200 mg/dL.

In our case, no specific cause was determined, and as described by Babic et al. in 2012 [7], chylous ascites was suspected to be due to lymphatic vessel rupture caused by pelvic congestion associated with pregnancy, due to hormonal progesterone-induced vasodilation and increased intrabdominal pressure from the gravid uterus.

The published literature provides very limited information on the management of this condition during pregnancy. Currently, dietary interventions are among the most commonly employed therapeutic options due to their safety. In specific and selected situations, pharmacological measures can also be considered as part of the treatment. Treatment modalities are aimed at providing symptomatic relief, with a focus on addressing the underlying etiology [1].

If the patient exhibits optimal oral tolerance, the first step is to remove lipids from the diet, as lipids consist mainly of long-chain triglycerides (LCTs) in 95%, which increases lymphatic flow. For this reason, supplementing the diet with medium-chain triglycerides (MCTs) is recommended, as these lipids are transported through the portal system.

If there is inadequate response to dietary measures, in cases where oral diet or enteral nutrition is not feasible, or if drainage is excessive, the use of parenteral nutrition is suggested. In this case, lipids are delivered directly into the bloodstream, thereby not contributing to chyle formation. Parenteral nutrition should be maintained for a period of 4 to 6 weeks to achieve clinical improvement [14].

Regarding pharmacological measures, the use of somatostatin or its synthetic analog, octreotide, is recommended. The latter has shown benefits in closing chylous fistulas. The mechanism of action is not well understood, but it is proposed to be multifactorial: it suppresses the exocrine function of the pancreas and reduces splanchnic flow, and therefore, the absorption of fats.

There is insufficient data in the literature regarding the safety of pharmacological therapy during pregnancy. The therapeutic needs of each patient should therefore be individualized.

Analyzing the documented cases in the literature, it has been observed that termination of pregnancy allows for complete resolution of chylous ascites. However, further studies are needed to conclusively determine if termination of pregnancy is the definitive treatment for this condition.

## 4. Conclusion

Chylous ascites is a very rare event during pregnancy. Its etiology is still not clearly understood. Although it is usually a benign condition that resolves spontaneously in the postpartum period, it often requires the termination of the pregnancy. It is important for healthcare providers to be aware of this condition in order to provide timely medical care and administer appropriate treatment.

## Figures and Tables

**Table 1:** Cases of chylous ascites in pregnant women reported to date.

Reference	Case	Year	Database	Symptoms
Thompson and Al Khabbaz [2]	Incidental chylous ascites at the time of cesarean section	2015	Case Reports in Obstetrics and Gynecology	Preeclampsia Vaginal bleeding Mild abdominal pain
Ul Ain et al. [3]	Idiopathic chylous ascites simulating acute appendicitis: a case report and literature review. Literature review	2016	International Journal of Surgery Case Reports	Lower abdominal pain Nausea
Apikotoa and Wijesuriya [4]	Idiopathic acute chylous peritonitis during pregnancy, mimicking perforated acute appendicitis: a case report	2021	International Journal of Surgery Case Reports	Right lower quadrant abdominal pain Fevers Vomiting
Burgos Luna et al. [6]	Quiloperitoneo espontáneo en el embarazo	2020	Revista Colombiana de Cirugía	Absent fetal movements Headache Epigastralgia Arterial hypertension
Zhang et al. [5]	Idiopathic chylous ascites in pregnancy: a case report	2018	Iran J Public Health	Abdominal distension and pain after vaginal delivery
Babic et al. [7]	Spontaneous resolution of chylous ascites following delivery: a case report	2012	Journal of Medical Case Reports	Asymptomatic
Arslan et al. [9]	Idiopathic chylous ascites following vaginal delivery: a case report	2019	Academic Journal of Gastroenterology and Hepatology	Nausea and vomiting Abdominal distension Tenderness
Yang et al. [8]	Acute hyperlipidemic pancreatitis accompanied by chylous ascites with normal amylase and lipase in pregnancy	2017	Journal of Clinical Lipidology	Nausea Vomiting Fever Epigastric tenderness
Buils-Vilalta et al. [10]	Laparoscopic management: spontaneous chyloperitoneum in pregnancy	2017	The Journal of Surgery	Diffuse abdominal pain Abdominal tenderness
Habek et al. [11]	Nontraumatic chyloperitoneum in pregnancy	2005	European Journal of Obstetrics & Gynecology and Reproductive Biology	Asymptomatic Accidentally discovered during cesarean section
Sun et al. [12]	A rare case of pregnancy complicated by mesenteric mass: what does chylous ascites tell us?	2007	World Journal of Gastroenterology	Asymptomatic finding in ultrasound

## Data Availability

All data are already included in the manuscript.

## Supplementary Materials

Supplementary Materials The rest of the case data belong to the patient's clinical history, and no other supplementary material is available.
