# Supplementary figures and images for: Acute Abdomen in Pregnancy due to Idiopathic Chylous Ascites
Source: Case Rep Obstet Gynecol. 2024 Feb 29;2024:8898451. doi: 10.1155/2024/8898451 (PMC11390214; doi:10.1155/2024/8898451)

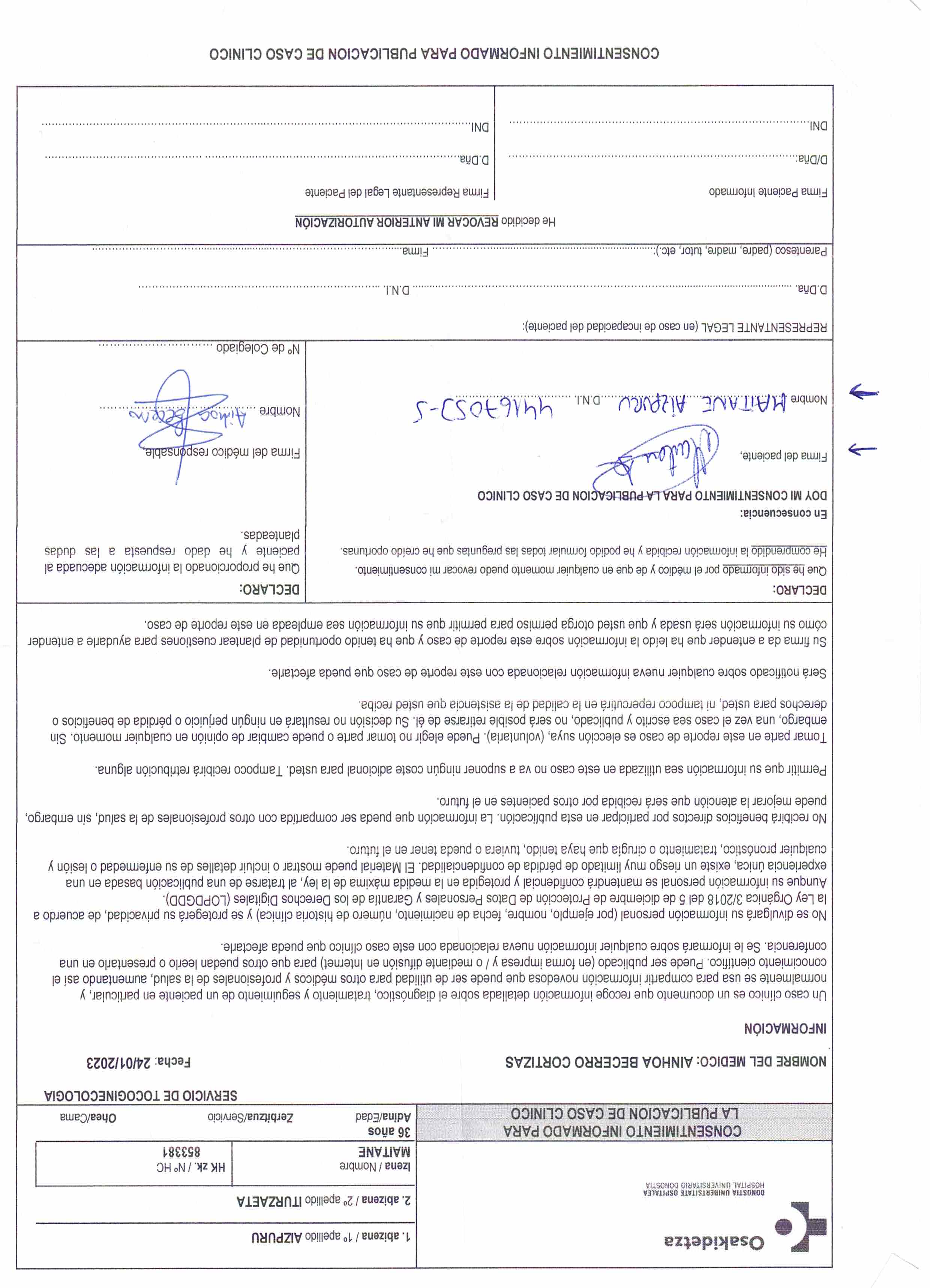

Supplement: Supplementary Materials — The rest of the case data belong to the patient's clinical history, and no other supplementary material is available. [file 8898451.f1.jpg]
